# Supplementary material for: High-throughput measurement of elastic moduli of microfibers by rope coiling
Source: Proc Natl Acad Sci U S A. 2024 Mar 13;121(12):e2303679121. doi: 10.1073/pnas.2303679121 (PMC10962939; doi:10.1073/pnas.2303679121)
Supplement: Supplementary file 1 — Appendix 01 (PDF) [file pnas.2303679121.sapp.pdf]

PNAS

Supplementary Information for:

High-throughput measurement of elastic moduli of microfibers by rope coiling

Yuan Liu[a,1], Jack H.Y. Lo[b,1], Janine K. Nunes[c], Howard A. Stone[c,2] & Ho Cheung Shum[a,2]

[a] Department of Mechanical Engineering, The University of Hong Kong, Hong Kong SAR, China.

[b] Centre for Integrative Petroleum Research, King Fahd University of Petroleum and Minerals, Dhahran, Saudi Arabia.

[c] Department of Mechanical and Aerospace Engineering, Princeton University, Princeton, New Jersey, USA.

[1] These authors contributed equally.

[2] Corresponding authors: Howard A. Stone & Ho Cheung Shum

Email: hastone@princeton.edu; ashum@hku.hk

This PDF file includes:

Supporting text

Figures S1 to S9

Legends for Movies S1 to S4

Other supporting materials for this manuscript include the following:

Movies S1 to S4

Setup and flow profile

The coiling device is made by connecting two glass capillaries to form a small channel upstream (560 μm wide) and an abruptly enlarged wide channel downstream (1746 μm wide). The small channel is a circular tube, while the wide channel is a square tube (to minimize image distortion), as shown in Fig. S1 below. The flow velocities on the different planes are calculated via COMSOL. Our setup and the flow are approximately axisymmetric. To show the downstream velocity more clearly, we replot the data with a narrower range in Fig. S2.

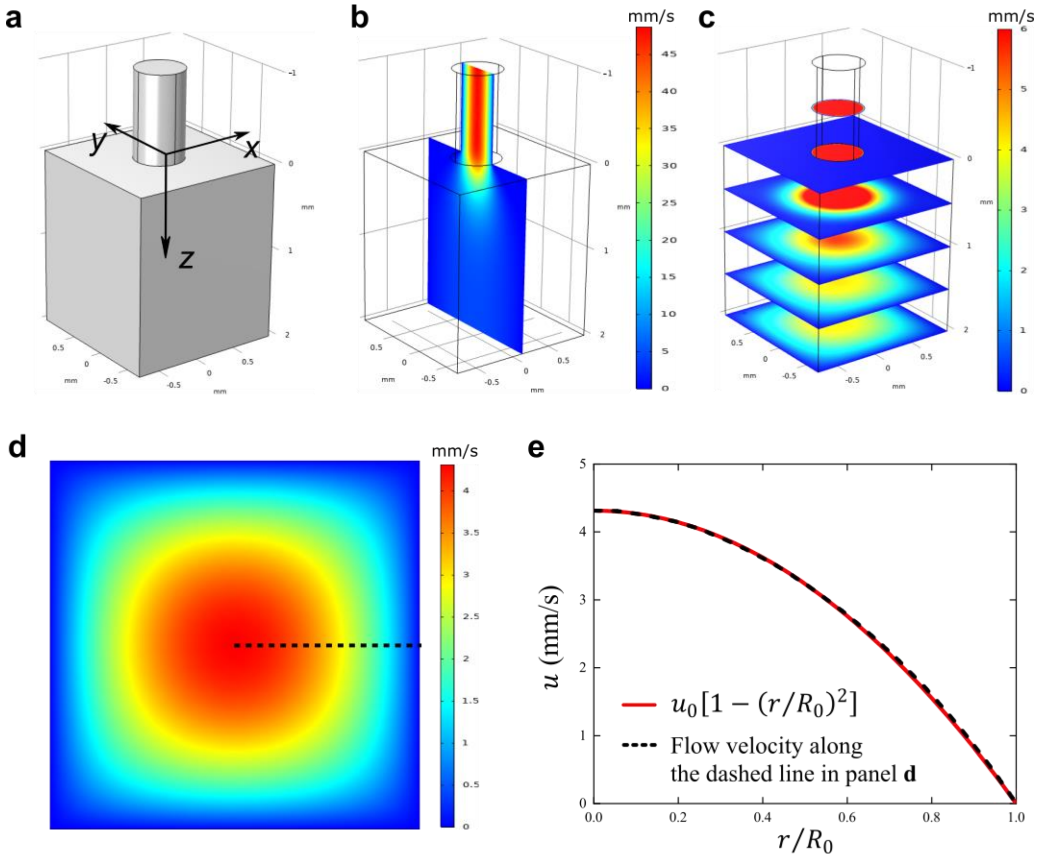

Figure. S1. Setup and the flow. a, Coiling device consists of a small circle tube and a wide square tube. **b**, Flow velocity on the yz plane at x=0. **c**, Flow velocity on the xy planes at different z. **d**, Flow velocity on the xy plane at *z*=1.5 mm. **e**, Flow velocity profile *u* along the dashed line of panel **d**, which is a parabolic function of radial position.

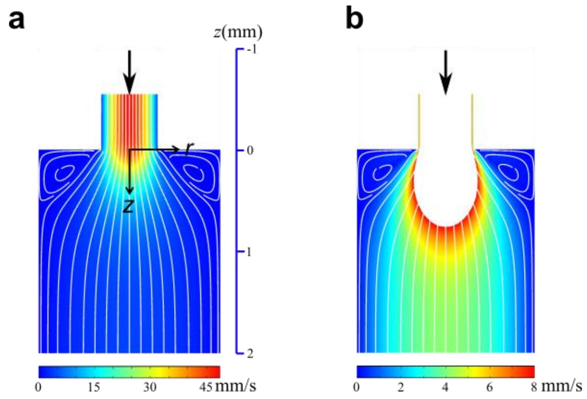

Figure. S2. Flow velocity and streamlines. a, Calculated velocity profile in the coiling device, same as Fig. 2*B* in the main text. **b**, Same data replotted in the low velocity region.

Flow velocity in different wide channels

We calculate the flow velocities and streamlines via COMSOL for different wide channels R_0=580 μm and 873 μm respectively, as shown in Fig. S3.

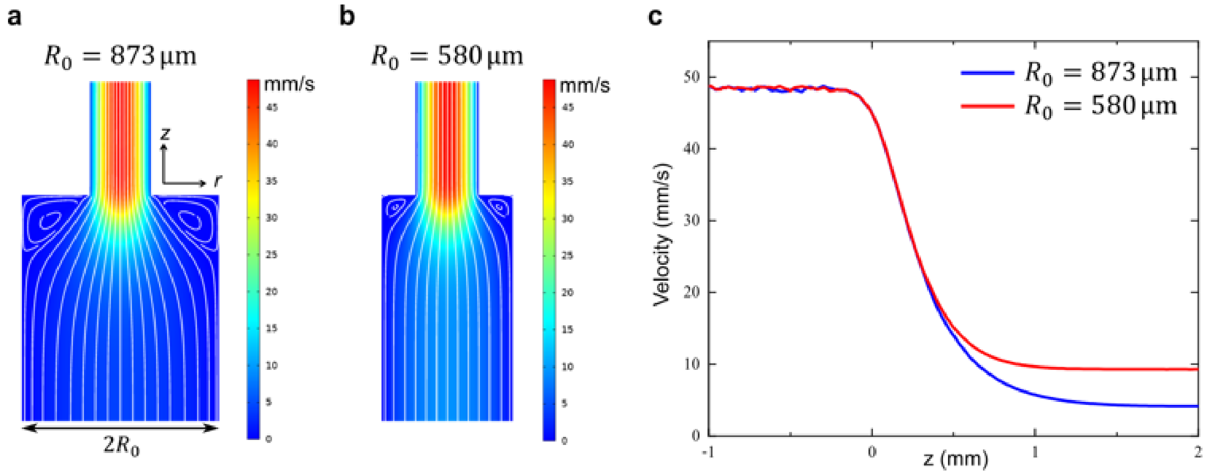

Figure. S3. Calculated flow velocities. a and **b**, The calculated velocity and streamlines for R_0=580 μm and 873 μm respectively. **c**, Plot of the velocity in **a** and **b** alone the central line r=0.

Elastic modulus measurements of tensile tester

The elastic moduli of microfibers are measured by a tensile tester (Agilent Technologies T150). The elastic moduli are obtained from the slopes of the measured stress-strain curves, as shown in Fig. S4 below.

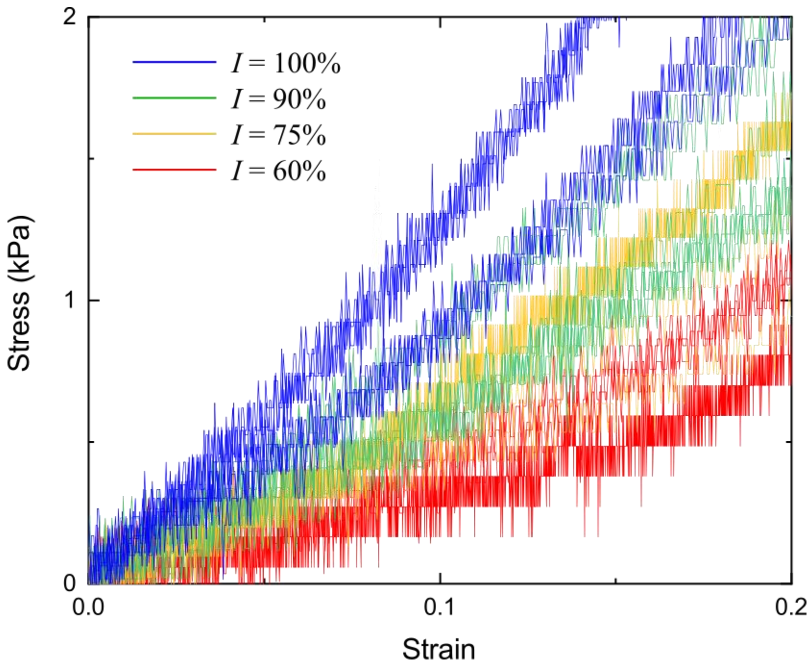

Figure. S4. The strain-stress curves of microfibers with different elastic moduli by adjusting different UV intensities. The legend shows the corresponding intensities of UV light, *I*, given in percentage of relative strength.

The absence of plastic deformation and viscoelastic effects

From the stress-strain curve, we can show that the fiber has not reached its proportionality limit during coiling: First, we selected two extreme samples with maximum strain of 0.11 and 0.5, corresponding to the maximum and minimum elastic moduli in all experiments. The maximum strain of coils is estimated as $\frac{2\pi(R+r)}{2\pi R} - 1 = \frac{r}{R}$, where R and r are the coiling radius and the fibers' radius respectively. Next, we perform the tensile test on these two fibers beyond the maximum strain calculated above. From the stress-strain curves, as shown in Fig. S5 below, we can see that there is no sign of plastic deformation.

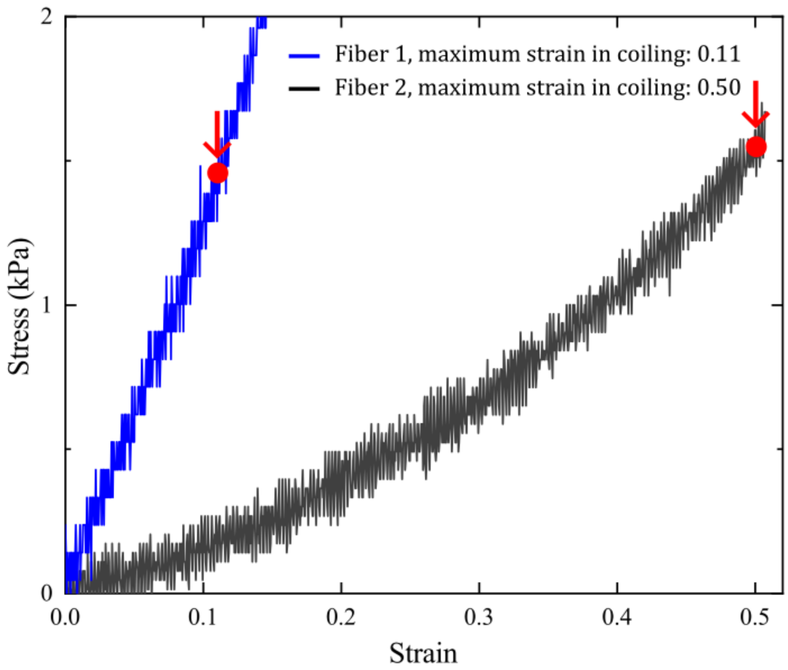

Figure. S5. The stress-strain curves of two representative microfibers. Red dots mark the maximum strain during coiling, which is 0.11 for fiber #1 and 0.50 for fiber #2.

Regarding viscoelastic effects, we conducted the stress relaxation test at constant strain as shown in Fig. S6 below. The final strain of 0.15 is reached at ~40 sec. No noticeable stress relaxation occurred during the 220 sec measurement period, indicating that the relaxation time is much longer than 3 minutes. In contrast, the time scale of the coiling is just ~0.1 sec, much shorter than the relaxation time. Therefore, in our experiment, the fibers behave as purely elastic.

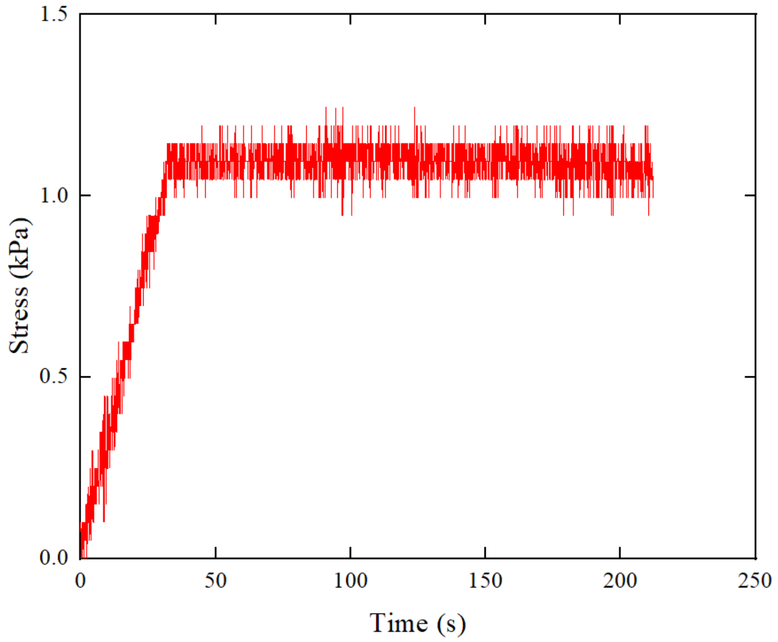

Figure. S6. The stress-versus-time response of microfiber. The fiber strain is 0.15.

Buckling of fiber

We find that the shape of buckling of our experiment resembles the buckling of a column with one end fixed and one end pinned, as shown in the figure below.

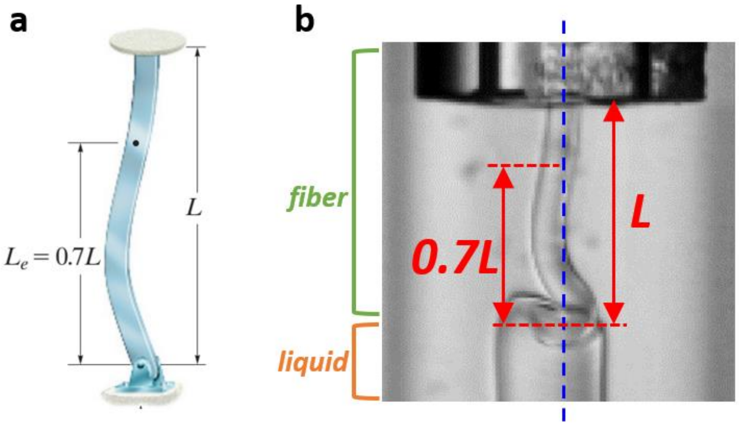

Figure. S7. a, A typical drawing of a buckling column with one end fixed and one end pinned. (Adapted from R.C. Hibbeler, Pearson, 10th Ed) **b**, Snapshot of the fiber buckling. L is the buckling length.

Fiber with axially varied elastic modulus

1.

The elastic modulus, E, of a fiber increases with the intensity of the UV light, I, used in the production. Inspired by Duprat et al [Lab Chip, 2015, 15. 244], the relation $E(I)$ is represented by:

$$E(I) = Ae^{\lambda(\frac{D}{v})\sqrt{I}} + C$$

where D is the diameter of the UV spot, v is the flow velocity. The parameters A, λ and C are deduced from curve fitting based on the data from different fibers, each with a different elastic modulus while other conditions remain the same, as shown in Fig. S8c. The red line is the best fit.

2.

A fiber with axially varied elastic modulus along its length is prepared by increasing the intensity of the UV light linearly with time during fiber production, as shown in Fig. S8a below and represented by:

$$I(t) = \frac{\Delta I}{\tau} t + I_c$$

The profile of the elastic modulus for this non-homogenous fiber is calculated by

$$\tilde{E}(s) = \frac{1}{\Delta t}\int_{t_s}^{t_s+\Delta t} E(I)\, dt = \frac{v}{D}\int_{s/v}^{(s+D)/v} E(I)\, dt$$

where s is the path distance from the soft end, as shown in Fig. S8b.

It is calculated numerically by integration with D=2mm, v=42mm/s, I_c=35, ΔI=65, τ=0.3s, and plotted in Fig. 3D (solid line) and Fig. S8d below.

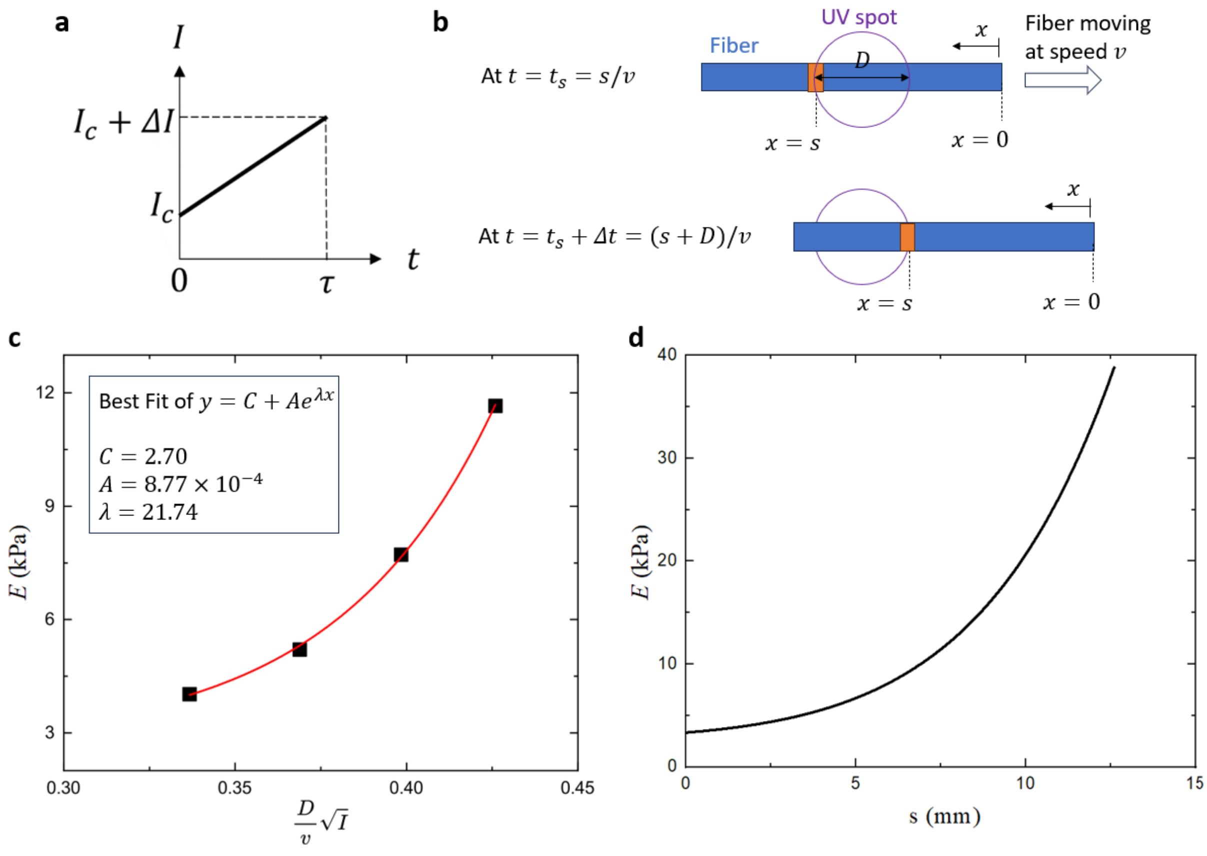

Figure. S8. Elastic modulus of a non-homogenous fiber axially varied by increasing UV intensities linearly with time.

Speed of coil measurement

The speed of coil v is obtained by measuring the distance Δz travelled by a coil within a finite time Δt, as shown in Fig. S9 below.

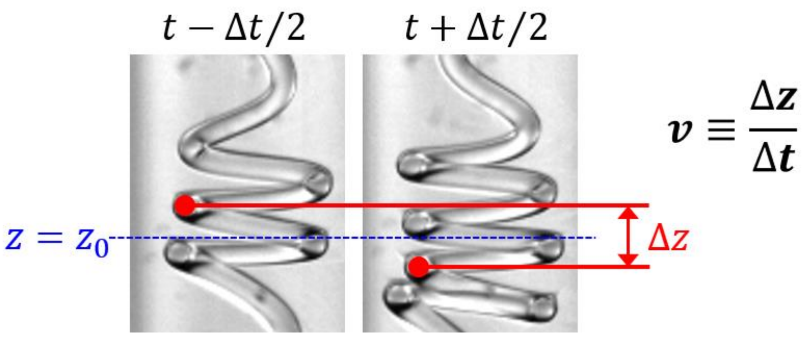

Figure. S9. Illustration of the coil speed measurement.

Supplementary Movies:

Movie S1. High-throughput elastic modulus measurement of microfibers. Microfibers with different elastic moduli enter the channel continuously at a high throughput of 3000 fibers per hour. Coiling occurs spontaneously, the resulting geometry is influenced by the elastic modulus. The device can run continuously for at least 90 min. This video captures 20 fibers as an example. The scale bar is 500 μm.

Movie S2. Demonstrating the reproducibility of coiling by continuous injection of fibers with same size and same elastic moduli. The throughput is 3300 fibers per hour. The scale bar is 500 μm.

Movie S3. A side-by-side movie showing the coiling of microfibers with different elastic moduli. As the elastic modulus increases from left to right, the coiling radius increases. The scale bar is 500 μm.

Movie S4. Coiling of a microfiber with axially varied elastic moduli. The coiling radius is small at the soft end, and large at the rigid end. The scale bar is 500 μm.
